# Effects of iron-deficient diet on sleep onset and spinal reflexes in a rodent model of Restless Legs Syndrome

**DOI:** 10.3389/fneur.2023.1160028

**Published:** 2023-05-18

**Authors:** Sydney Woods, Joseph Basco, Stefan Clemens

**Affiliations:** Department of Physiology, Brody School of Medicine, East Carolina University, Greenville, NC, United States

**Keywords:** RLS, inducible phenotype, reversible phenotype, mouse, sleep behavior, spinal reflexes

## Abstract

Restless Legs Syndrome (RLS) is a common sensorimotor and a sleep disorder that affects 2.5–10% of the European and North American populations. RLS is also often associated with periodic leg movements during sleep (PLMS). Despite ample evidence of genetic contributions, the underlying mechanisms that elicit the sensory and motor symptoms remain unidentified. Clinically, RLS has been correlated with an altered central iron metabolism, particularly in the brain. While several animal models have been developed to determine the outcome of an altered iron homeostasis on brain function, the potential role of an altered iron homeostasis on sleep and sensorimotor circuits has not yet been investigated. Here, we utilize a mouse model to assess the effects of an iron-deficient (ID) but non-anemic state on sleep time and episodes, and sensorimotor reflexes in male and female mice. We found that animals on the ID diet displayed an increased expression of the transferrin receptor in the spinal cord, confirming the results of previous studies that focused only on the impact of ID in the brain. We also demonstrate that the ID diet reduced hematocrit levels compared to controls but not into the anemic range, and that animals on the ID diet exhibited RLS-like symptoms with regard to sleep onset and spinal cord reflex excitability. Interestingly, the effects on the spinal cord were stronger in females than in males, and the ID diet-induced behaviors were rescued by the return of the animals to the control diet. Taken together, these results demonstrate that diet-induced ID changes to CNS function are both inducible and reversible, and that they mimic the sleep and sensorimotor RLS symptoms experienced in the clinic. We therefore propose replacing the commonly used phrase “brain iron deficiency” (BID) hypothesis in the RLS research field with the term “iron deficiency in the central nervous system” (ID-CNS), to include possible effects of altered iron levels on spinal cord function.

## 1. Introduction

Restless Legs Syndrome (RLS) is both a sensorimotor and a sleep disorder characterized by uncomfortable sensations deep in the legs (“urge to move”). These sensory symptoms are often accompanied by periodic leg movements during sleep (PLMS). RLS has a prevalence of anywhere between 2.5–10% in the Europe and North America, depending on severity (1, 2), and a hallmark of RLS symptoms is that come with a circadian component and usually peak in the evening and at night (3–6). RLS can manifest itself as either idiopathic or secondary RLS, and both versions express the same symptomatology. However, while secondary RLS generally resolves after resolution of the underlying triggers, the causes for idiopathic RLS are unknown or non-obvious and the symptoms usually do not resolve, leading to generally life-long symptoms.

RLS is familial in about 50% of patients (7), and genome-wide association studies (GWAS) have implicated over 20 risk loci in the genome that increase the likelihood of developing idiopathic RLS (8–15). In response to the GWAS studies, several genetically modified animal models have been developed to interrogate the impact of these genes on RLS. A comparison of these different knockout models for BTBD9, MEIS1, MAP2K5, triple-opioid receptor, and dopamine (DA) D3 receptor identified several overlapping phenotypes that correspond to RLS in the clinic (e.g., an increase in overall activity, restlessness, and locomotion) (16). However, the assessment of sleep-related aspects was not addressed uniformly and relied on wheel running (17), motor behaviors (18), or EEG and EMG electrodes that were tethered to the animals (19).

RLS responds well to initial treatment with dopaminergics that target the inhibitory D2-like receptor family [e.g., (20–23)], suggesting a functional role of dopamine (DA) system in the disorder (24). At the same time, long-term treatment with these dopaminergics is also regularly associated with a gradual worsening of the symptoms [augmentation; (25–29)], indicating that the D2-like receptor system may interact with other mechanisms as well (30–33).

There is strong evidence that RLS is associated with alterations in intracerebral iron availability due to undefined defects in iron homeostatic mechanisms (25, 34–38). Based on these findings, several animal models have been developed that assess how alterations to systemic or brain-specific iron levels modify behavior and/or correlate with the clinical symptoms [summarized in; (16)]. Specifically, studies on recombinant inbred BXD mice have demonstrated distinct sex-related differences and distinct biological rhythms in the regulation of iron content (39, 40) that also affect the DA system (41) and that are in line with changes in clinical symptoms (42). In addition, a brain iron deficient phenotype has also been developed by exposure to iron-deficient diet upon weaning (42–45).

However, none of these studies explored the behavioral effects of altered iron levels on spinal reflexes or characteristics of sleep such as onset, number of episodes, or duration. Therefore, we here used this diet-induced iron deficient (ID) model (43) to probe for the effects of this inducible RLS animal model on these sleep parameters and on thermal pain withdrawal reflexes in the spinal cord.

## 2. Methods

### 2.1. Animals

All experimental procedures were approved by the East Carolina University Institutional Animal Care and Use Committee and were fully compliant with the National Institutes of Health guide for the care and use of Laboratory animals (NIH Publications No. 80–23). All efforts were made to minimize the number of animals used. A total of 72 C57Bl/6 mice (males and females) was used for the experimental parts of the study. To establish the diet phenotypes, lactating C57Bl/6 dams and their litters were obtained from Charles River (strain 026, Raleigh, NC) at postnatal days 10–12 and housed in standard cages with food and water available *ad libitum*, with a 12 h light/dark cycle and at a temperature of ~20° C. Upon weaning (postnatal day 21), the offspring was separated from their mothers, housed in male and female pairs, and provided with iron control and iron deficient diets, respectively.

### 2.2. Diet

**Figure 1:**
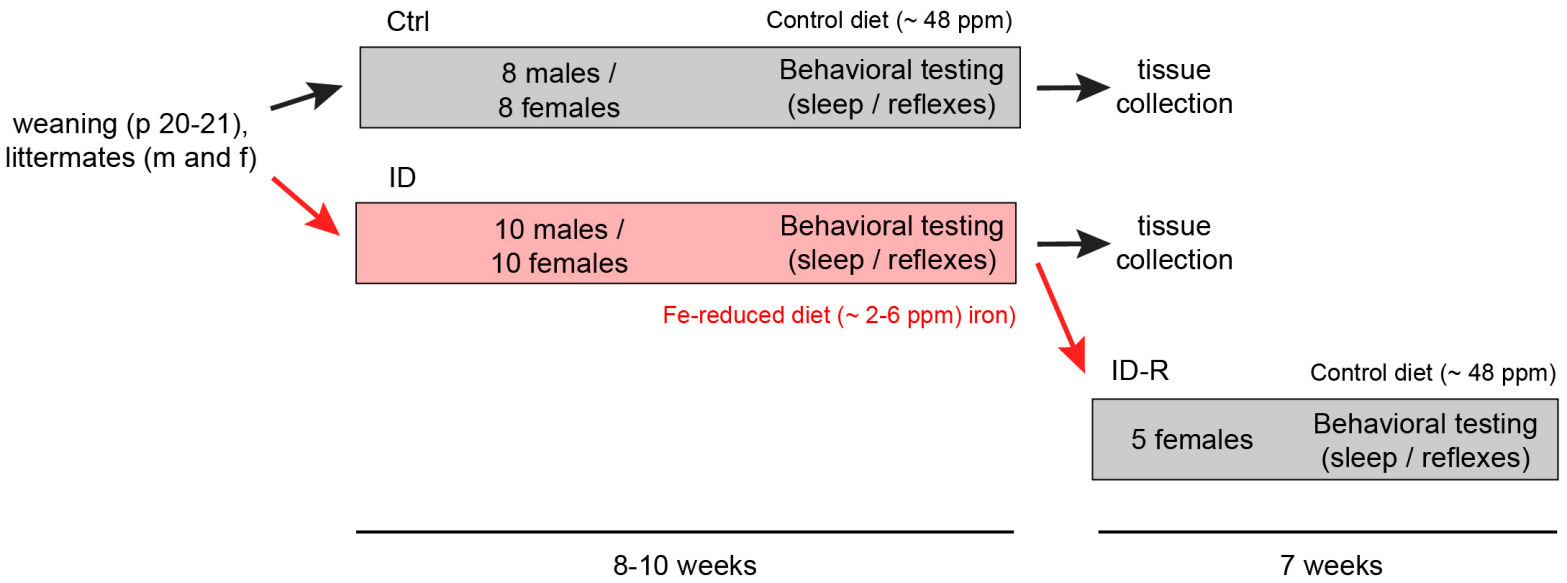
Flowchart of induction and reversal of iron deficiency (ID) and experimental layout. C57Bl/6 mice were separated upon weaning into cohorts fed control iron (Ctrl, ~48 ppm Fe, 8 males and 8 females) and cohorts fed iron-reduced diets (ID, ~2–6 ppm Fe, 10 males and 10 females). At the end of the sleep studies, one ID cohort (ID-R, *n* = 5) was subsequently placed on normal diet to assess potential recovery from the ID phenotype as assessed in the thermal pain withdrawal reflex latencies. Thermal pain withdrawal reflex latencies were assessed throughout the initial separation and toward the end of the ID-R recovery period. Sleep studies were performed in the last 2–3  weeks of initial exposure and the last week of recovery.

Following previously established protocols (43), weaned animals were fed either with iron-control diets (Control, Envigo Teklad TD.99398, ~ 48 ppm Fe; 14 males and 22 females) or iron-reduced diets (ID, Envigo Teklad TD.99397, 2–6 ppm Fe; 14 males and 22 females). Both diets are a modification of the standard TD.80394 and TD.80396 diets, respectively, with added vitamin levels to make diets more suitable for irradiation. After 10 weeks of exposure, a subset of female mice on the ID diet (N = 4) was returned to the control diet for 7 weeks to assess for the potential recovery from the iron-deficient diet (ID-R). A flowchart of the experimental protocol is shown in Figure 1.

### 2.3. Assessment of systemic iron status

To assess if the ID diet led to an anemic phenotype, blood samples were taken from a subset of animals at time of sacrifice to measure serum hematocrit levels using a standard Readacrit Micro-Hematocrit centrifuge system (Clay-Adams, Becton, Dickinson & Co, Parsippany, NJ), and the data collected were then transformed to calculate hemoglobin levels.

### 2.4. Plessey sensors for non-invasive movement detection in the home cage

Four commercially available electric field (EF) sensors (Plessey Semiconductors, PS25251 1 cm2, +/− 5 V) were provided to us by the group of Shawn Hochman (Emory University School of Medicine) (46). In short, these EF sensors measure changes in the local electric field within their detection area caused by movement and translate it into a voltage trace (+/− 5 V, 2048 Hz sampling rate). When attached to the outside of the cage, the EF sensors detected large and small mouse motions with high temporal resolution and record them as electric field disruptions. The corresponding voltage transients relate to the amplitude of the movements (e.g., large transients for rearing, and small transients for resting respiration) (46). One pair of EF sensors was fitted to each electrically-separated compartment of the testing cage, to allow for the sleep recordings of up to two animals simultaneously. The signals from the EFs were filtered through a low-pass filter at 30 Hz, digitized with a Digidata 1440A using pClamp software (Molecular Devices, Sunnyvale, CA), and stored off-line for analysis in Spike 2 (Cambridge Electronic Design, Cambridge, United Kingdom).

### 2.5. Sleep observations

The test cage was placed inside a darkened Faraday cage with a red LED strip background light (light intensity inside the Faraday cage: < 100 Lux). Each cage compartment contained a water and food distributor, to allow for *ad libidum* feeding and drinking during the 5-h sessions. A video camera (Logitech C920 HD Pro, Logitech, Newark, CA) was set up in front of the cage to record the animals’ activities and correlate them with the EF recordings. Animals were placed in the compartments at 8 am in the morning and EFs and behavior were recorded in epochs of 1 h for up to 5 h. After the end of the 5-h recording sessions, animals were returned to their home cage and taken back to the animal room.

### 2.6. Sleep analysis

Following data acquisition, sleep epochs recorded with pClamp were imported into Spike 2 software (Cambridge Electronic Design, Cambridge, UK) and converted in sonogram mode (46). The top dB value was set at 38, the range dB value at 33, and block size was 4,096, in a Hanning configuration. Sleep was determined by video analysis of 60 s of continuous stillness of the animal with no discernable movement (47) and the lack of large-scale movement-related excursions in the EF recordings (Supplementary material, Video #1). After 60 s of no visual movement on the video and no large fluctuations on the EF sensors, we considered the animal asleep and started recording sleep time. Animal movements during sleep were identified by large excursions in the EF signals and verified with the parallel video recordings. To differentiate short-lasting movements during sleep from other movements, such as scratching or adjustments in the nest, we set the duration limit for movements during sleep at 1 s. This allowed us to clearly distinguish these short-lasting movements (“twitches”) from all other behaviors (i.e., head movements, full-body adjustments, grooming or other non-twitch behaviors, Supplementary material, Video #2). To quantify differences in sleep behaviors, we plotted data in terms of individual hours per day, from the first through the fifth hour. The behavior of each animal was recorded for 2–5 subsequent days, and data from these days were averaged per animal to calculate the following parameters per hour: sleep time, number of sleep episodes per hour, and duration of sleep episodes per hour.

### 2.7. Thermal pain withdrawal reflex testing

A Hargreaves IITC Plantar Analgesia Meter (IITC Inc., Woodland Hills, CA) was used to assess thermal pain reflex withdrawal latencies as a function of the diets in a subset of animals as previously described (32, 33, 48). In short, animals were weighed and placed into the individual acrylic enclosures on top of an elevated glass platform. These experiments were performed in a dedicated room under low-light conditions. After an acclimation phase of 1 h, animals were tested 5 times per session, with trial intervals of 5–7 min for each animal. Testing was usually performed 3–5 times per week between the hours of 8 am and 11 am, to minimize the potential impact of circadian fluctuations. Once initiated, recording sessions for all 5 trials lasted no longer than 60 to 90 min on any given day.

### 2.8. Tissue collection and protein quantification

Following the behavioral experiments, animals were humanely anesthetized with isoflurane and decapitated. Spinal cords were carefully dissected out, immediately placed in RNAlater (Thermo Fisher Scientific, Waltham, MA), and stored at −20° C until processing. To assess protein concentrations, tissues were homogenized in 1 mL of RIPA buffer with protease and phosphatase inhibitors (0.12 mL/mL RIPA buffer, Sigma-Aldrich, #P2714, and 0.012 mL/mL of RIPA buffer, Sigma-Aldrich, #P5726, St. Louis, MO, respectively). The homogenized tissues were centrifuged (13,000 rpm, 4° C, 15 min), and supernatants were aliquoted and stored individually at −20° C. Following homogenization, standard protein concentrations were established with a BCA protein assay (BCA protein assay kit, Thermo-Fisher #23227), and plates were read on an Epoch Microplate Spectrophotometer (BioTek, Winooski, VT) at 562 nm using the Gen5.1 software package (BioTek, Winooski, VT).

### 2.9. Western blots

For Western blots, 20 μg of each lumbar spinal cord protein samples were denatured using 2x Laemmli buffer containing 5% β-mercapto-ethanol and 1% SDS at 95° C for 10 min, loaded onto a 12% Criterion™ TGX Stain-Free™ Protein Gel (#5678045, Bio-Rad, Hercules, CA), and run for ~45 min at 200 V (Bio-Rad Power PAC 200, Hercules, CA). Protein gels were imaged with a transferred to a low fluorescent PVDF membrane using Trans-Blot® Turbo™ RTA Midi LF PVDF Transfer Kit (#170–4,275, Bio-Rad, Hercules, CA). Membranes were probed with the primary antibodies and with secondary antibodies (LI-COR, Lincoln, NE) on an iBind™ Flex Western Device (SLF2000, Thermo Fisher, Waltham, MA). Membranes were imaged with an Odyssey detection system (Odyssey Clx, LI-COR, Lincoln, NE) and associated software (Image Studio, Li-Cor), and analyzed with ImageJ software.

### 2.10. Antibodies

The primary antibodies used for Western blot to detect transferrin receptor-specific protein expression in the spinal cord was the anti-transferrin receptor (Abcam 84,036, 1:500 Cambridge, MA). The secondary antibody used was a goat anti-rabbit IRDye 800CW (926–32,211, 1:8,000, LI-COR Biosciences, Lincoln, NE). Transferrin receptor protein expression bands were normalized to protein expression on the entire length of the membrane.

### 2.11. Statistical analysis

Following the experiments, behavioral data were transferred and stored in Excel format, then analyzed and plotted offline with SigmaPlot (Version 11, SPSS Science, San Jose, California). For statistical comparisons, we employed parametric or non-parametric comparisons as appropriate when comparing multiple groups (One-Way ANOVA, RM ANOVA, or ANOVA on Ranks) with appropriate post-hoc comparisons; *t*-tests or paired t-tests were used for comparison between two sets of data (treatment against respective control vehicle treatment). Significance levels were set at *p* < 0.05.

## 3. Results

### 3.1. Effects of ID diet on hemoglobin levels

**Figure 2:**
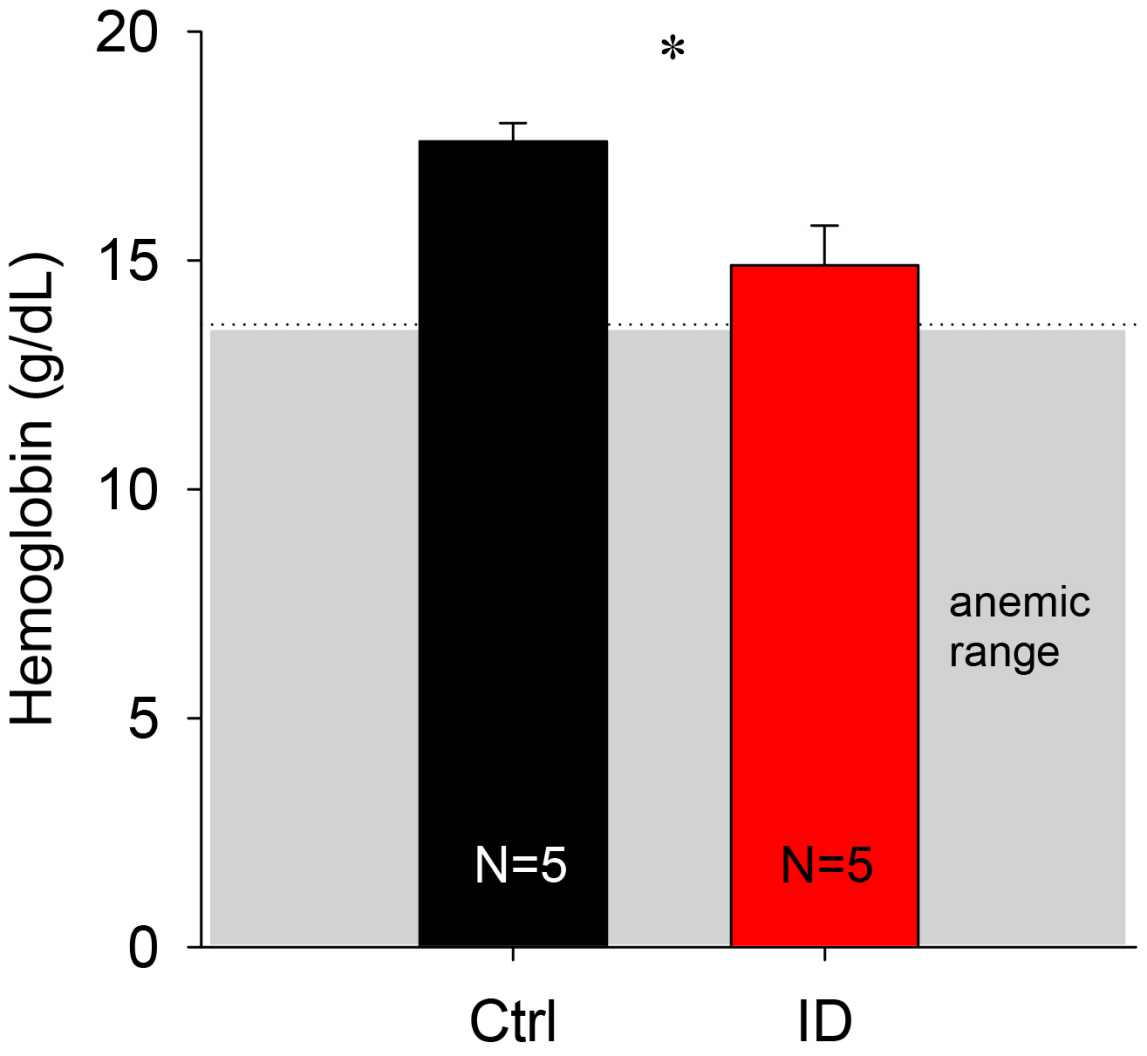
Comparison of calculated hemoglobin levels in animals on control and ID diets. We did not observe any significant difference between the two groups. Gray box indicates range of anemia.

To confirm that the iron deficient (ID) diet did not induce an anemic phenotype, we first measured hematocrit levels in the blood of a subset of animals fed with the ID diet at time of sacrifice. We chose to use hematocrit as it is a convenient and commonly used measure of anemia in humans (Figure 2). Animals fed the control diet expressed hematocrit levels of 52.7 +/− 2.8%, whereas the animals on the ID diet had slightly lower hematocrit levels of 44.6 +/− 2.5%. These hematocrit data translate to hemoglobin levels of 17.6 +/− 0.4 g/dL in the control animals and 14.9 +/− 0.9 g/dL in the animals on the ID diet. While hemoglobin levels in the animals on the ID diet were lower than the levels in the control animals (*p* = 0.023, *t*-test, power: 0.637, *N* = 5 per group), they remained above levels considered to represent an anemic phenotype in mice [13.9 g/dL and 13.6 for males and females, respectively (49)].

### 3.2. Effects of ID diet on transferrin expression levels in the spinal cord

**Figure 3:**
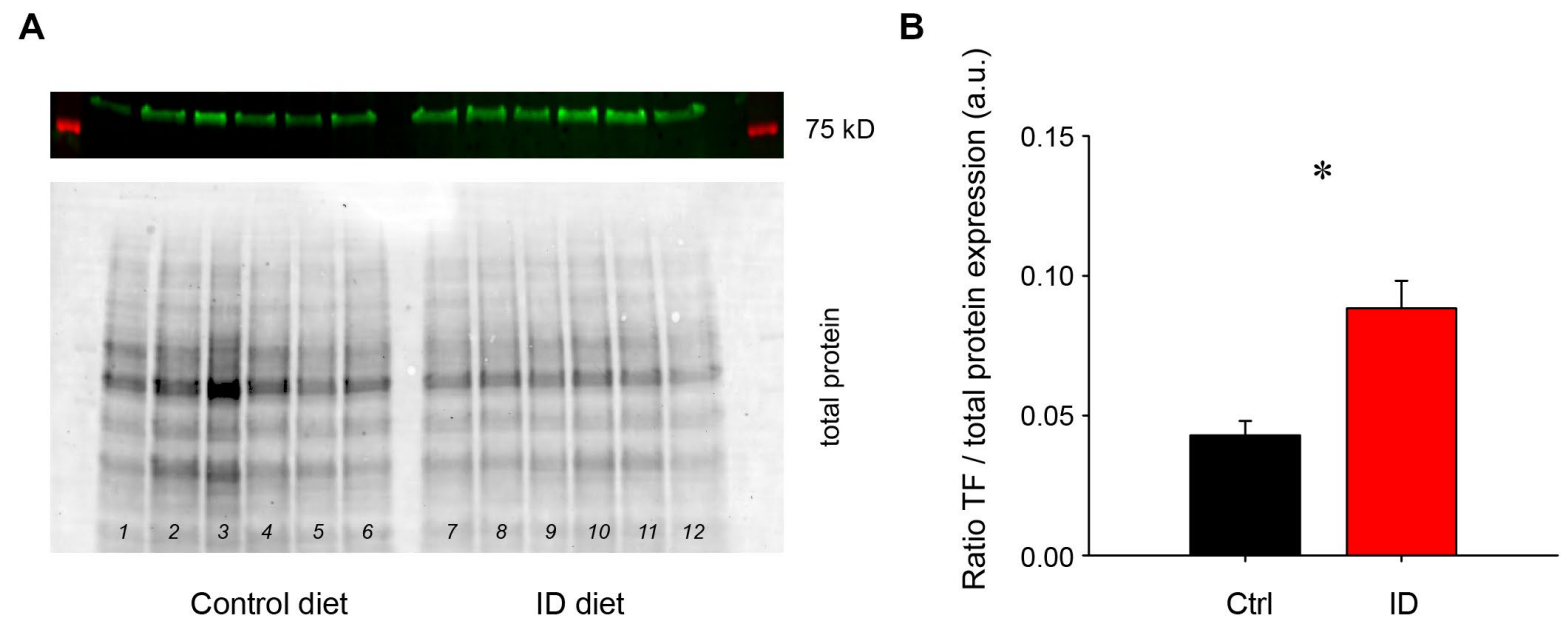
Protein expression levels of the transferrin receptor in lumbar spinal cords of animals on control and ID diets, normalized to total protein expression. **(A)** Transferrin (TF) receptor expression in animals on control diet (top panel), and their respective total protein expression (bottom panel). Lanes 1–6 represent lumbar spinal cords from animals on control diet, while lanes 7–12 represent tissues from the lumbar spinal cord of animals fed with the ID diet. **(B)** Quantification of the protein expression data. TF receptor protein expression normalized to overall protein expression was significantly increased in the spinal cords of animals on ID diet over those on the control diet.

It was previously shown that exposure to an ID diet led to an upregulation of transferrin levels in cortex and striatum (43). To test if the effects of low iron diet effects impact the spinal cord as well, we probed for transferrin levels in the spinal cord (Figure 3). We found that animals on the ID diet displayed a significantly higher ratio of the transferrin receptor in the spinal cord than animals on the control diet (Control diet: 0.043 +/− 0.01; ID diet; 0.088 +/− 0.01, *p* = 0.002, power: 0.956, *t*-test, *N* = 6 each). These data suggest that the spinal cord is as affected in a similar manner to the brain.

### 3.3. Effects of ID diet on overall sleep performance

**Figure 4:**
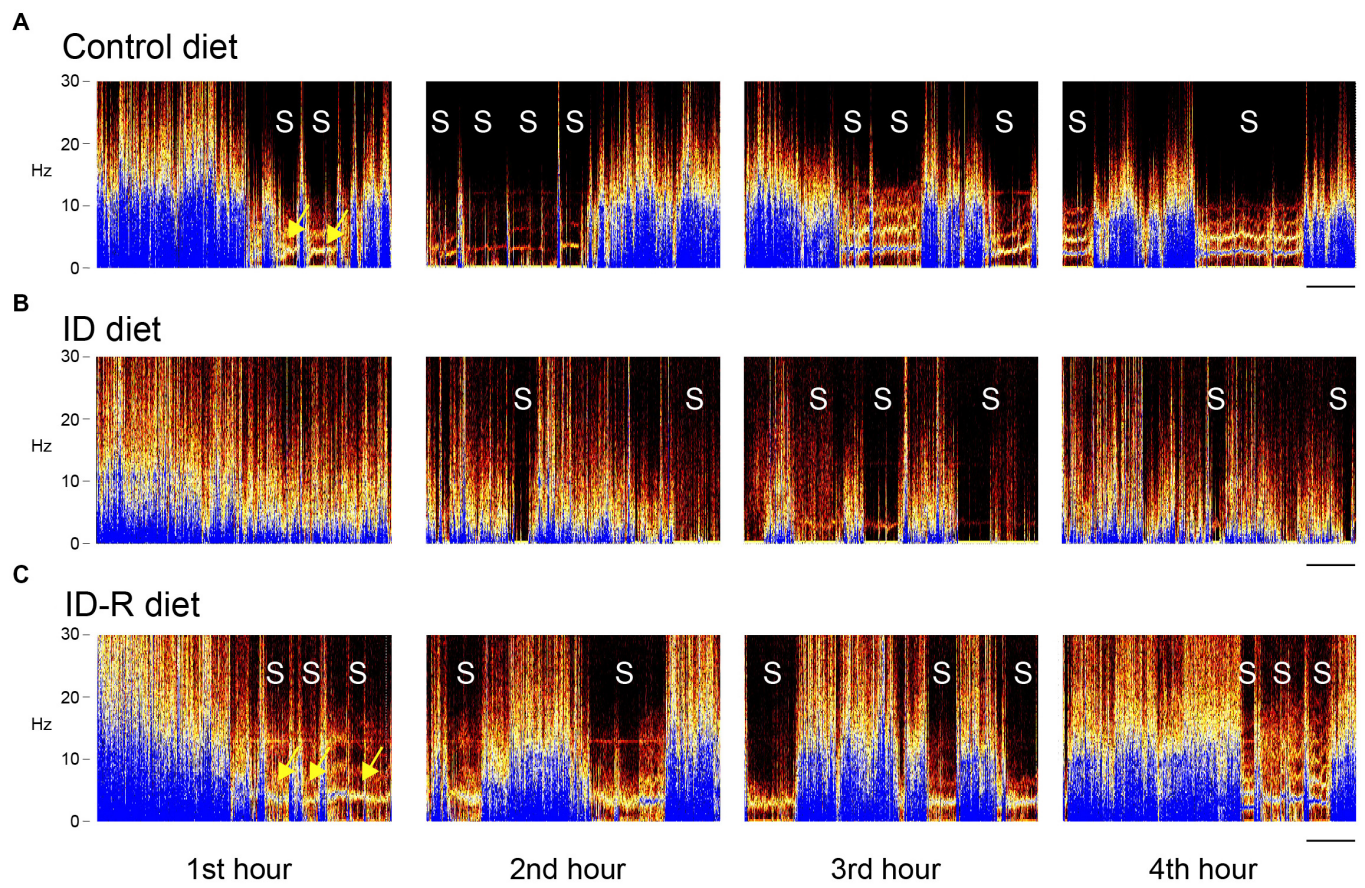
EF-derived activity spectrograms from animals on control diet, ID diet, or after return from the ID diet (ID-R) over 4 h of recordings. Activities in each block are plotted as frequencies (0–30 Hz) over time, and each block represents a 1 h-segment of a 4-h continuous recording session. **(A)** Representative recording of an animal on control diet. Over the 4-h period, the animal displayed a total of 11 sleep episodes (S), and these sleep episodes started as early as in the second part of the first 1-h segment. **(B)** Representative recording of an animal on ID diet. Over the 4-h period, the animal displayed a total of 9 sleep episodes (S), but none of these sleep episodes occurred during the first 1-h segment. **(C)** Representative recording of an animal on ID-R diet. The animal displayed a total of 12 sleep episodes (S), during the 4-h recording session, and, as with the animal on the control diet, these sleep episodes started as early as in the second part of the first 1-h segment. Yellow arrows point to the 4–5 Hz breathing band that can be detected when the animal does not move round in the cage. Scale: 10 min.

To determine if ID diets could modify the sleep pattern and leg movements in animals during sleep as observed in RLS patients, we tested the effects of ID diets on these behaviors during the initial 5 h of the animals’ sleep phase only, from 08 h00 a.m. to 13 h00 p.m., based on a light-ON/light-OFF schedule from 07 h45 a.m. to 19 h45 p.m. This approach allowed us to capture the animals’ activities in the early phase of their sleep cycles. The initial sonography analysis of the recordings provided a visual overview of rest and activity stages in animals on either control or ID diets (Figure 4). These representative spectrograms of animals on control diet, ID diet, or after return from the ID diet (ID-R) were obtained on the second day of recordings for each animal. Color intensity (dark to blue) denotes the power of respective frequencies present in the continuously recorded voltage trace from the EF sensors over a period of 4 h. During the first ~30 min of the first hour, EF recordings from all 3 animals display a wide range of frequencies, but only the animals on control and ID-R diet also displayed phases of reduced activity and sleep (S) during the first hour, during which the breathing-associated frequency band of ~4–5 Hz (50, 51) can be detected against the background (arrows). Sleep/resting phases are interrupted by very short bouts of leg movements (less than 1 s in duration) or slightly longer phases of that were associated with grooming or re-adjustments of the animal in its nest. During the second hour of recording, animals on all three diets showed periods of sleep, but those tended to occur more often and/or last longer in animals on Control diet or on Ex-ID diet (top and bottom panels) than in animals on the ID diet (middle panel). From the third hour on, EF and video recordings revealed generally similar behavioral phenotypes across all animals, regardless of their diet status.

Sleep studies were performed on 8 male animals on the control diet (Ctrl), 8 male and 2 female animals on the ID diet (ID), and 4 male animals after return from the ID diet for >7 weeks to the control diet (ex-ID). As we did not observe any significant difference between the 8 male and the 2 female animals on the ID diet, their data were grouped together in this and the subsequent analyses.

### 3.4. Effects of ID diet on accrued sleep time

**Figure 5:**
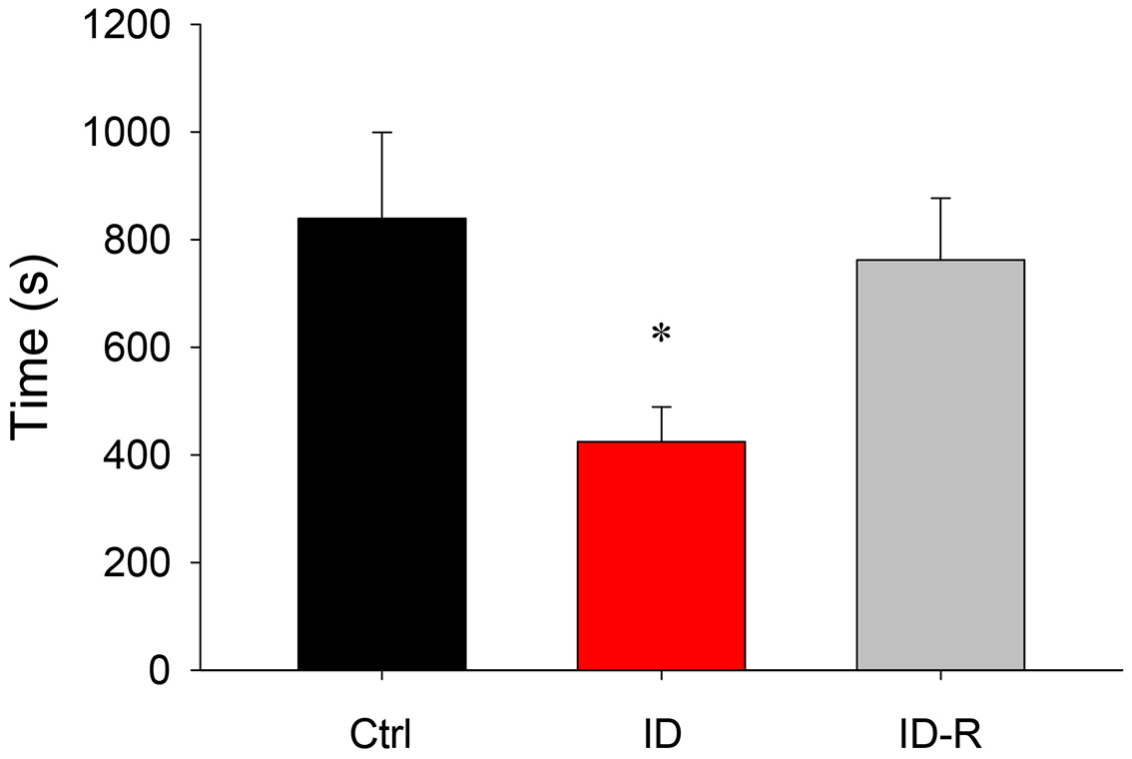
Overall hourly sleep times in animals on control, ID, and ID-R diets. Animals on the ID diet displayed significantly less time in sleep phases per hour than animals on control or ID-R diets.

A comparison of overall sleep time between animals on control, ID diet, and ID-R diet revealed that ID diet led to a significant but reversible decrease in sleep time per hour (Figure 5). Specifically, animals on the control diet slept on average 839 +/− 160 s/h (S.E.), animals on the ID diet slept on average 425 +/− 65 s/h, and animals on ex-ID diet slept on average 762 +/−115 s/h. The decrease in hourly sleep time in the animals on the ID diet was significant (*p* = 0.012, One-Way ANOVA). In parallel to the decrease in average sleep duration per hour, ID animals displayed a diet-induced and reversible decrease in the number of sleep episodes per hour. The average number of sleep episodes of 2.27 +/− 0.32 per hour in Ctrl animals decreased in animals on ID conditions to 1.59 +/− 0.19 per hour and returned in ex-ID to 2.26 +/− 0.29 per hour (*p* = 0.075, power: 0.33, One-Way ANOVA, data not shown).

### 3.5. Effects of ID diet on sleep episodes over time

**Figure 6:**
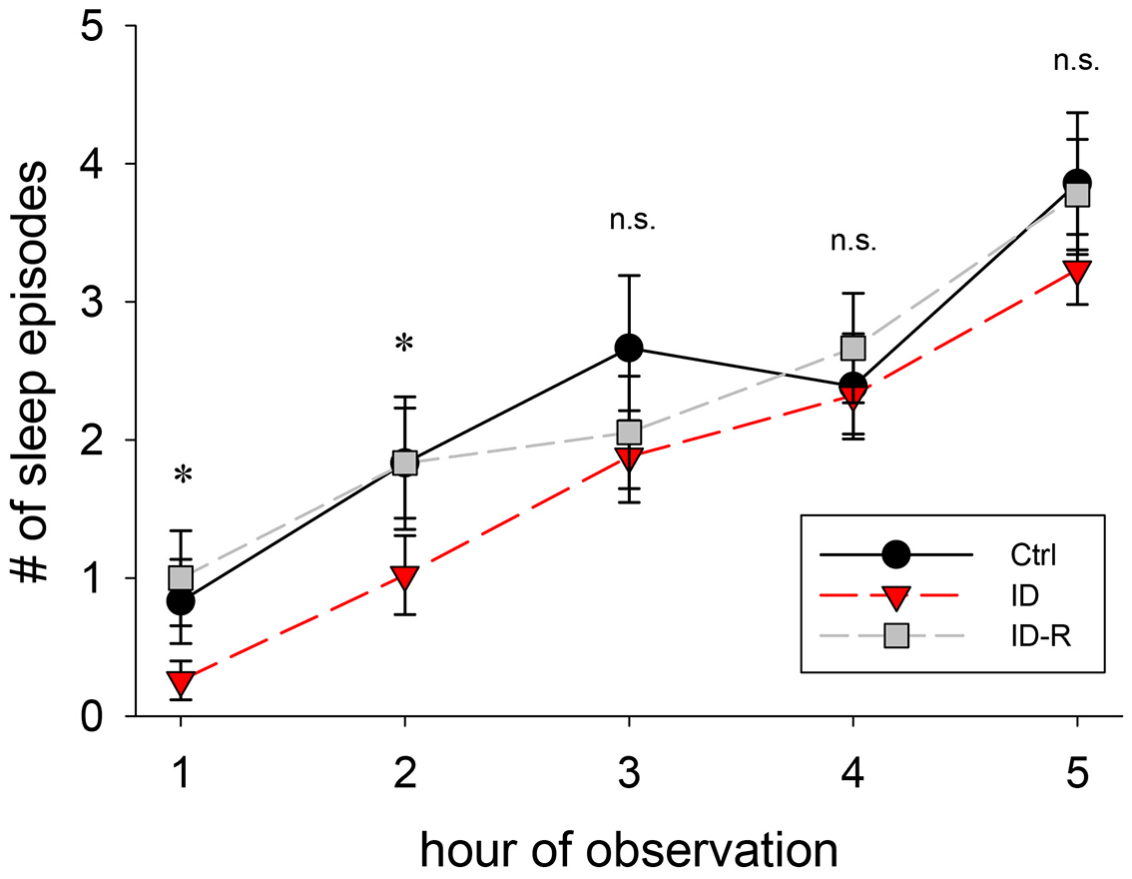
Number of sleep episodes per hour of observation. Animals on ID diet (red symbols) displayed fewer sleep episodes in the first and second hour than animals fed on control (black symbols) and ID-R diets (grey symbols). From the third hour on, all cohorts displayed a similar number of sleep episodes per hour.

RLS is clinically associated is with a difficulty of falling asleep rather than sleep itself, therefore we next analyzed the number of sleep episodes in the 3 animal cohorts as a function of the time during experimental sessions (Figure 6). We found that the number of sleep episodes per hour gradually increased in animals on control diet from 0.8 +/− 0.3 sleep episodes in the first hour to 3.8 +/− 0.5 episodes in the fifth hour. In animals on ID diet, we observed 0.4 +/− 0.5 sleep episodes in the first hour, which increased to 3.2 +/− 0.3 sleep episodes in the fifth hour. In animals after return to control diet, the number of sleep episodes rose from 1 +/− 0.3 episodes in the first hour to 3.8 +/− 0.4 sleep episodes in the fifth hour. There was a significant difference in the number of sleep episodes between Ctrl, ID, and ID-R during the first 2 h of recordings (1^st^ hour: *p* = 0.009; 2^nd^ hour: *p* = 0.042) but not in the subsequent hours. Thus, these data indicate the iron-deficient diet led to time-specific difference in sleep behaviors in the ID cohort that was reversible after return to normal diet.

### 3.6. Effects of ID diet on thermal pain withdrawal reflex latencies

**Figure 7:**
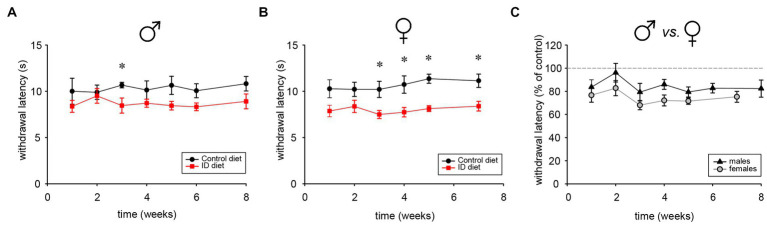
Thermal pain withdrawal latencies in animals on control, ID, and ID-R diets. **(A)** Development of withdrawal latencies of male cohorts fed on control (black symbols) and ID diet (red symbols), respectively. Significant differences are indicated by asterisks. **(B)** Development of withdrawal latencies of female cohorts fed on control (black symbols) and ID diet (red symbols), respectively. Significant differences are indicated by asterisks. **(C)** Comparison of withdrawal latencies of male (black symbols) and female (gray symbols) cohorts fed on ID diet. Data for each ID cohort were normalized to their respective group on control diet.

To determine the impact of the ID diet on spinal cord reflex latencies, male and female cohorts were tested repeatedly over time with the Hargreaves system. Starting as early as 1 week into the different diet regimen, we observed that males under the ID diet expressed slightly lower reflex latencies than those on the control diet (Figure 7A). After 3 weeks, reflex latencies became significantly different between Ctrl and ID cohorts, with values of the animals on the ID diet at about 80% of their respective controls (*p* < 0.001, One Way RM ANOVA, power = 0.99, N = 6 per group). In the female cohort, the effect of the ID diet mirrored the trend from the male animals, and thermal pain withdrawal latencies decreased to ~80% of the values obtained in the controls (p < 0.001, On Way RM ANOVA, power = 0.992, N = 6 per group) (Figure 7B). We next directly compared the effects of the ID diet in males and females with each other (Figure 7C). Throughout the testing period, females consistently displayed a slightly larger effect of the ID diet on their thermal pain withdrawal reflexes. From week 3 on, reflex latencies in males on ID diet ranged between 79 +/− 7.6% to 87 +/− 5.3% of those from their littermates on control diet at the same time point. In contrast, in the female animals on the ID diet, the corresponding data ranged from 68 +/− 3.9% to 75.3 +/− 4.7% of their littermate controls. However, while consistent, the difference between these data was not significant (*p* < 0.1, On Way RM ANOVA, power = 0.325, *N* = 5 for males and *N* = 4 for females). Together, these data indicate that exposure to the ID diet decreases spinal cord mediated withdrawal reflex latencies in both males and females to a similar degree, but that the effect appeared slightly stronger in females than in males.

### 3.7. Effects of return to normal diet on thermal pain withdrawal reflex latencies

**Figure 8:**
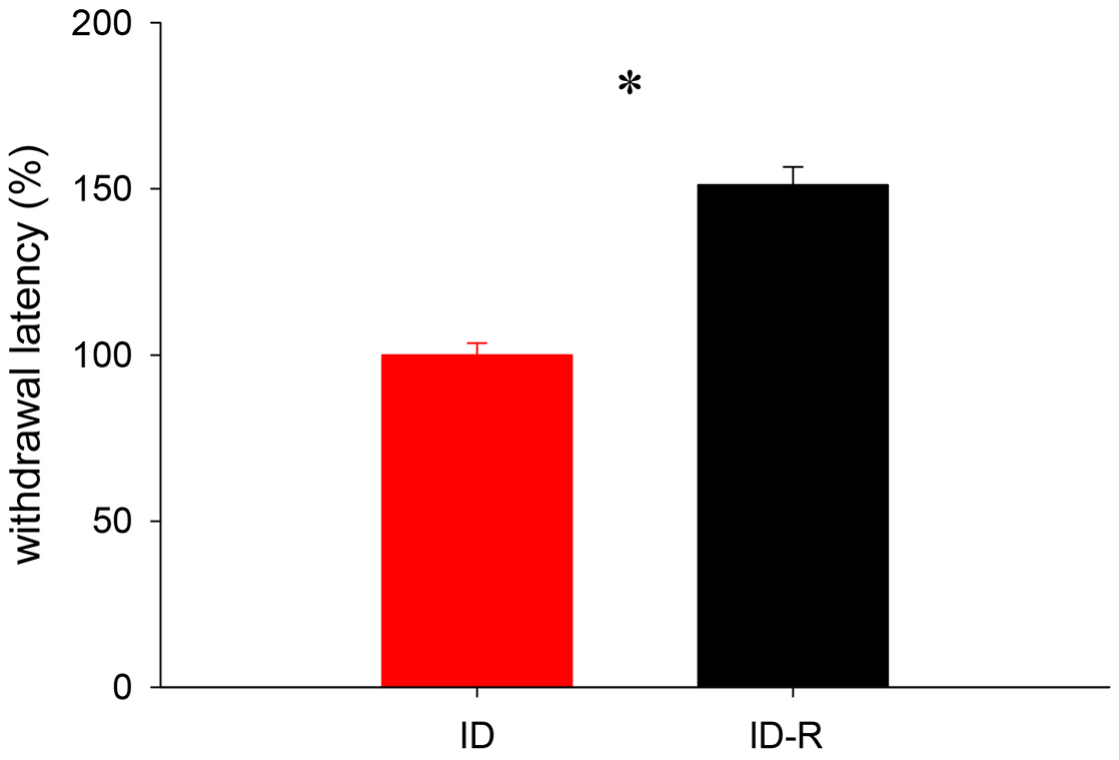
Assessment of thermal pain withdrawal latencies in animals on ID and after return to ID-R diet, normalized to data obtained under ID diet conditions. Reflex latencies in animals on ID-R diet were significantly increased over ID diet.

We next used a subset of female animals on the ID diet to test if a return to normal diet influenced reflex withdrawal latencies as well (Figure 8). We found that, after animals were switched back to the normal diet, reflex withdrawal latencies in this group increased after 7 weeks significantly, from 99.99 +/− 3.5% to 151.2 +/−54% (*p* < 0.001, *t*-test, power = 1.0, *N* = 5 per group). These data support the findings from the sleep study and they also suggest that the impact of the ID diet on thermal pain withdrawal reflex latencies is also both inducible and reversible.

## 4. Discussion

This study examined the effects of diet-induced iron deficiency (ID) on sleep and reflex behaviors in a mouse model of RLS. Our key findings are that exposure to ID diets led to a decrease overall sleep time and the number of sleep episodes in animals exposed to the diet. In addition, the ID diet also led a decrease in thermal pain withdrawal latencies, thus indicating a heightened sensorimotor excitability in the spinal cord. Importantly, these effects were reversible after return to normal diet. Our data suggest that ID diet affects both brain and spinal cord circuits and function, and that a return to a diet with normal iron levels can revert these changes.

RLS is a multifaceted neurological disorder with a wide range of both genetic and non-genetic components. The interplay between these different factors makes it difficult to identify a singular animal model that combines face validity with construct validity (52, 53). Several risk genes have been identified in genome-wide association studies, and the mutation of these risk gene orthologs in mice showed phenotypes relevant to RLS, such as alterations in iron metabolism, dopamine function, sensory perception, and sleep [e.g., MEIS1: (33, 54, 55); BTBD9: (56–58); PTPRD: (18); D3KO: (30, 33, 59, 60)]. However, it is possible that associated compensatory mechanisms may have contributed to the resulting phenotypes.

### 4.1. The impact of ID diet on peripheral iron levels

A common pathophysiological feature of RLS patients is an altered iron status in the brain (20, 61–64), and these alterations in iron homeostasis have been associated with altered dopamine function (36). It has been speculated that alterations in the iron management protein profile in RLS at the site of blood–brain interface underlie fundamental differences in brain iron acquisition (65). As anemia can be a contributing factor to RLS (66, 67), we here wanted to assess if our dietary protocol affected the peripheral system in the mice. We found that our ID protocol led to a small decrease in hemoglobin levels in the animals on the ID diet, but that those values remained above those considered anemic in mice (49). Our findings are also supported by earlier findings from a different lab using similar animal models (43, 68). We therefore postulate that the behavioral changes we observed with our approach arose from alterations in the CNS and not from the periphery.

### 4.2. The impact of ID diet on transferrin receptor expression in the spinal cord

A diet-induced ID status has been associated in the past with an increase in transferrin (TF) receptor expression in the striatum (43), underlining the importance of this protein in iron homeostasis. However, there have previously been no attempts to also probe for potential changes in TF receptor protein expression in the spinal cord. As the spinal cord is the ultimate gateway for the sensory inputs and motor outputs that play a role in RLS, we tested the impact of the ID diet protocol on TF receptor protein expression in the lumbar spinal cord. Our findings indicate that the spinal cord responds similar to the ID diet as reported previously for the striatum (43), thereby providing cause to test for functional changes in the spinal cord as a result of the ID diet. Recent studies have provided evidence that the spinal flexor withdrawal reflex may be clinically relevant to the pathogenesis of PLMS as experienced by RLS patients (69, 70). As the withdrawal reflex circuitry is polysynaptic, it is difficult to hypothesize which aeras of the spinal cord may be show a preferential effect to the effects of the ID diet, if any. Based on our findings that transferrin ratio is increased in the spinal cord in animals on the ID diet and that these animals exhibit heightened spinal sensorimotor excitability, one of our future directions for this project is to perform immunohistochemistry in the spinal cord to determine which structures may be predominantly affected by the ID diet.

### 4.3. The impact of ID diet on sleep

A hallmark feature of RLS is the difficulty or even inability of patients to fall asleep. Previous animal studies that assessed sleep in an animal on ID diet have reported a reduction in REM sleep time, increased sleep fragmentation, and the occurrence of tibialis anterior EMG bursts akin to human PLMS (71–73). While these approaches identified sleep-related changes in the respective RLS animal models, they did not analyze the initial hours of the rest phase that corresponds to the falling asleep phase. We here used a non-invasive approach to record locomotor, rearing, and breathing activities from freely behaving animals in the first 4–5 h of their sleep phases, using electrical field (EF) sensors. These EF recordings in turn were corroborated and validated by parallel video recordings (46). Our data show that the effect of the ID diet reduces the number of sleep episodes in the first 2 h of the rest phase only, but that the behavior between littermates on control diet, ID diet, or ID-R diet is indistinguishable at later time points. This indicates that the effects of the diet-induced ID have a temporal component that is most pronounced at the equivalent of the falling asleep-phase in humans.

### 4.4. The impact of ID diet on spinal cord reflexes

RLS symptoms are defined as the urge to move the legs, and this urge is usually accompanied or caused by uncomfortable and unpleasant sensations in the legs. This urge to move the legs and the leg movements implies a role for the spinal cord circuits in mediating and relieving RLS symptoms. Women have a higher prevalence for developing RLS (35, 74, 75), but the current genetic animal models did not assess potential sex differences (16). Diet-induced ID has been shown to modify increase acute and chronic pain responses and male mice, possibly by altering to the acute pain threshold and sensitivity to C-fiber-mediated chronic pain in ID RLS patients (76), but there, the effects of the diet on female counterparts were not studied. Here, we show that exposure to the ID diet led to larger effects in female than in male littermates. This suggest that this simple interventional approach may provide a reversible tool with which to decipher the sex-specific mechanisms that mediate the excitability of the spinal cord as a function of its own iron homeostasis. We are currently performing pharmacological experiments that are aimed to decipher the differential responsiveness of these circuits to select drugs in males and females.

### 4.5. Brain iron deficiency vs. CNS iron deficiency

A large body of work has analysed the relationship between iron and RLS [reviewed in: (16)], and multiple clinical studies have highlighted an association between iron storage and function and RLS symptomatology [e.g., (61, 75, 77)]. The focus on changes to the iron status in the brain has led to the development several animal models in which the role of ID was assessed in either diet-induced ID models (44, 71, 78–81), or through selective breeding in BXD recombinant inbred mice (40, 82). This focus on brain-related iron deficiency has led to the emergence of the term “brain iron deficiency” (BID) in th eliterature (42, 45). Our data in this study show that diet-induced ID has effects well beyond the brain, in that it changes transferrrin rexeptor expression in the spinal cord and that reversibly alters the excitability of spinal cord reflex circuits. We therefore propose to replace the term BID in the RLS literature with the more appropriate term “iron deficiency in the central nervous system” (ID-CNS).

## Data availability statement

The raw data supporting the conclusions of this article will be made available by the authors, without undue reservation.

## Ethics statement

The animal study was reviewed and approved by East Carolina University Institutional Animal Use and Care Committee.

## Author contributions

SW, JB, and SC contributed to conception and design of the study, analyzed the data, and wrote sections of the manuscript. SW and JB acquired the data. SW and SC performed the statistical analysis. SW wrote the first draft of the manuscript. All authors contributed to manuscript revision, read, and approved the submitted version.

## Funding

This work was supported by the Department of Physiology and the Brody School of Medicine at East Carolina University (SC).

## Conflict of interest

The authors declare that the research was conducted in the absence of any commercial or financial relationships that could be construed as a potential conflict of interest.

## Publisher’s note

All claims expressed in this article are solely those of the authors and do not necessarily represent those of their affiliated organizations, or those of the publisher, the editors and the reviewers. Any product that may be evaluated in this article, or claim that may be made by its manufacturer, is not guaranteed or endorsed by the publisher.
